# Influence of soil physical and chemical characteristics on soil compaction in farm field

**DOI:** 10.1016/j.heliyon.2024.e25140

**Published:** 2024-01-23

**Authors:** Yared Seifu Woldeyohannis, Someshakher S Hiremath, Simie Tola, Amana Wako

**Affiliations:** a Department of Mechanical Engineering, DDU, Ethiopia; b Department of Mechanical Engineering, IIT Madras, India; c Department of Mechanical Engineering, ASTU, Ethiopia

**Keywords:** Farm field, Soil chemical properties, Soil compaction, Soil physical properties

## Abstract

Farm soil compaction is influenced by animal loads and Agricultural machinery. In this paper the influence of soil physical and chemical characteristics on soil compaction at Awash Melkasa farm field. Compaction of soil test was taken at five different depths which are; 5 cm, 10 cm, 15 cm, 20 cm, and 25 cm with the help of a hydraulically operated cone penetrometer. Those five depths were used in 15 sample points (point A to point O) to take 75 soil compaction data using hydraulic powered a Spot-on digital soil cone penetrometer from an area of 0.6 ha farmland. A correlation of 15 sample points (A to O) of soil compaction in the field was performed. For soil physical and chemical tests in laboratory soil samples were taken from selected farm fields at 3 different ranges of depths (0–10, 10–20, and 20–30 cm). Averagely the highest and the lowest compaction values are 3947.32 Kpa and 2667.72 Kpa respectively. The soil texture laboratory test indicates the soil was a clay loam with 36.74 % sand soil, 30.31 % clay soil, and 33 % silt soil. The highest and the lowest percentages of moisture value were 13.97 and 16.04 respectively. Total organic carbon, organic matter, and total nitrogen increase as the soil compaction increases and vice versa. The output of this study adds value to the field of agricultural mechanization since the weight of machinery is high, knowing the soil's physical and chemical properties and investigating the relation with the soil compaction rate is necessary.

## 1 Introduction

In recent farm soil-related research areas most of them are worried about farm field soil compaction as farm tractors and field machinery weight become increased [1]. In advanced agricultural mechanization, agricultural machinery is used from primary tillage operation to post-harvesting processes by driving them in farm fields [2]. The tillage concentration and tractor wheel load increase the bulk density and the increase of organic matter inside the soil decreases the bulk density [3]. Related research also indicates the weight of agricultural machinery compacts the soil increases the soil bulk density, and reduces soil porosity and crop yields [4]. Farm field soil compaction is also influenced by animal loads rather than agricultural machinery in developing countries. In developing countries, soil compaction is also influenced by tillage methods and crop rotations [5]. Soil tillage method, crop rotations in single farm fields, the water content of soil, and tillage depth are the main concerns in soil management and agroecosystem areas [6]. Because of its increased strength and draft resistance while being compacted, the soil is challenging to plow. The supporting capacity, transport, preservation, and availability of water and nutrients to plants are all dependent on the physical characteristics of the soil concerning farming, ecological, and engineering applications [7].

Compaction of soils in agricultural farm fields is commonly in the past research measured traditionally using the help of hand power to insert the soil cone penetrometer [[8], [9], [10]]. In tillage research, the soil cone index is frequently used to characterize the soil's physical qualities and assess soil strength. Even soil compaction is used in maintaining a good root environment during rainy and windy environments it also has side effects in reducing aeration and water penetration and increases runoff [11]. Cone index values vary with soil depth, textural characteristics, bulk density, and moisture content. According to studies done on farmland, the values of the soil cone index increase as soil clay, sand, and silt content increase [12].

Soil organic carbon (SOC) content in Enset farming, which is employed in climate-resilient technologies, is greatly influenced by Wolka, Biazin [13]. Soil physical features include the movement of water, air, and dissolved compounds, as well as conditions that influence germination, root growth, and erosion processes [14]. The common soil physical and chemical properties affecting the soil compaction are soil texture, soil salinity (pH), soil organic matter (SOM), soil total nitrogen (STN), soil electrical conductivity (SEC), soil organic content (SOC) and soil cation exchange capacity (SCEC [15,16]. The moisture content of the soil was measured in different ways, like electrical devices that are equipped with radioactive chemicals [17]. Generally, the soil moisture content of the soil is found with the numerical relation of the weight of moist soil to the dry soil in a particular mass of soil [18,19]. The study conducted on Prosopis growing farmland shows Prosopis planting soils had a massive effect in increasing the soil pH and exchangeable sodium percentage [20]. Mihretie et al. researched for three years to investigate the influence of tillage practice of row planting and broadcast planting methods on soil physiochemical properties [21].

Recently the advancement of agricultural mechanization has resulted in an increase in soil particles to denser which results in difficulty in air and water circulation [22]. Most of the time soil compaction was measured with the help of a manual-operated cone penetrometer which is time-consuming as addressed in the related studies keeping in mind some researchers use a mechanically pushed soil cone penetrometer to measure soil compaction [23]. Based on the past studies gap this research paper investigates soil physical and chemical properties like; moisture content, soil PH level, soil clay, silt, sand percentage, SEC, SCEC, SOC, SOM, and (STN) that influence soil compaction in the study area.

## 2 Materials and methods

### 2.1 Description of the study area

**Fig. 1:**
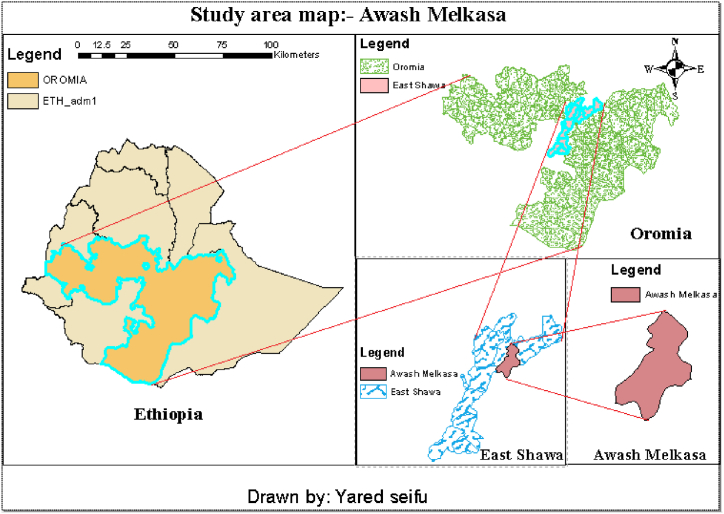
Study area map.

In this research farm site was Awash Melkasa for both soil compaction tests and soil chemical and physical laboratory tests. The experimental site is located in one of Ethiopia the biggest regions of Oromia. This study location is found at an elevation between 1400 and 2500 m above sea level [24]. The location of the study area map drawn by ArcGIS is indicated in Fig. 1. The farm field selected was (60 m by 100 m) total with an area of 6000 m^2^ and was conventionally tilled approximately for more than 25 years.

### 2.2 Experimental design for soil compaction measurement

**Fig. 2:**
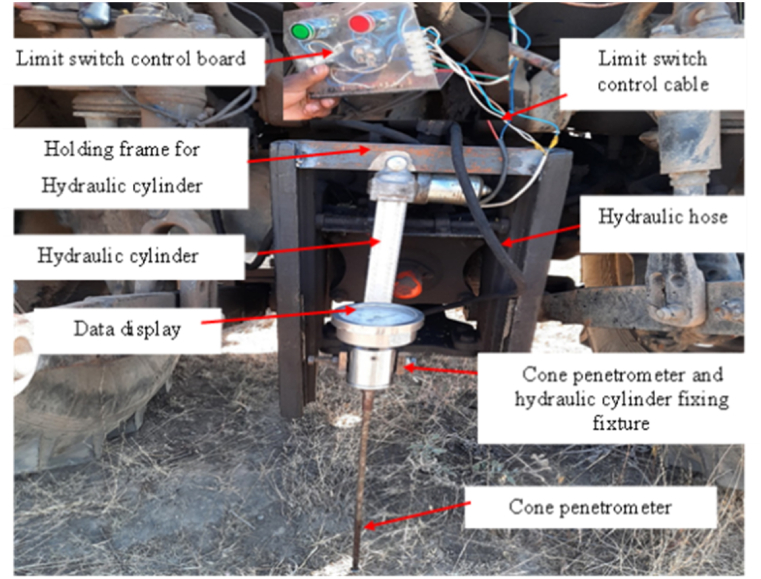
Cone penetrometer and tractor integration.

Compaction soil test was taken at five different depths which are; 5 cm, 10 cm, 15 cm, 20 cm, and 25 cm with the help of a hydraulically operated cone penetrometer. Those five depths were used in 15 sample points (point A to point O) to take 75 soil compaction data using hydraulically powered a Spot-on digital soil cone penetrometer. As indicated in Fig. 2 the cone penetrometer fixture was fixed to the at the tip of the piston rod. The circuit from the limit switch cell was connected to the hydraulic cylinder system through a voltage excitation circuit. The limit switch cell was energized from the battery of the tractor through an inverter and the excitation circuit was powered by an external 24V battery. Before conducting field experiments, the pressure and flow rate were adjusted by manual method. The lever of the direction control valve was operated to give forward movement to the penetrometer and the limit switch controls the depth of operation. Just after completion of the taking of data the hand lever and the limit switch were operated in reverse direction to retract the piston to its original position.

### 2.3 Physical and chemical property of soil

**Fig. 3:**
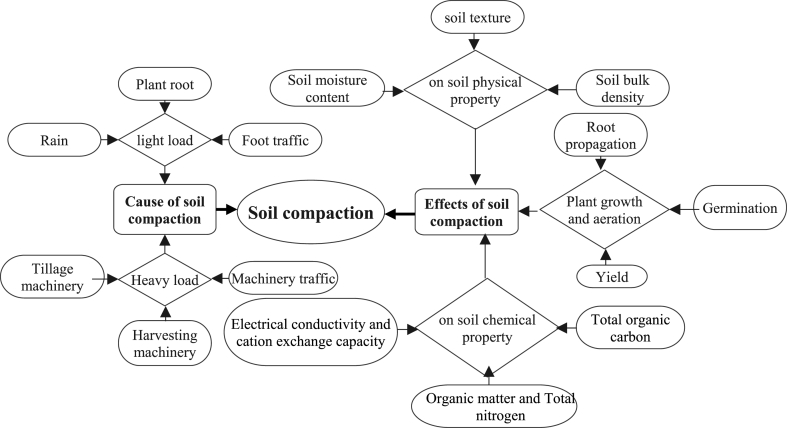
Cause and effects of soil compaction.

The causes and effects of soil compaction parameters are indicated by the flow chart Fig. 3. The experimental area selection criteria are based on the tractor density and availability of the research center. The field under study had a size of 0.6 ha and was tilled for more than 25 years.

The physiochemical characteristics of soil were determined in a laboratory after taking soil samples from the ranges of depths (0–10 cm, 10–20 cm, and 20–30 cm). The soil physical characteristics laboratory test addressed soil texture and moisture content. Also, soil chemical characteristics like pH value, SEC, SCEC, SOM, STN, and SOC are determined in a laboratory. Using a container, an electric oven, and a digital scale, the moisture content of farm field soil was determined. Soil moisture content is determined with the help of (Equation (1)) after the before and after weight test.

(1) $$w\left(\%\right)=\frac{\left(w_{1}+w_{2}\right)-w_{3}}{w_{3}}\times 100$$

Where; *W* = Moisture in (%)

*W*_*1*_ = Container's weight in kg.

*W*_*2*_ = Weight of container plus moist soil in kg.

*W*_*3*_ = container weight + dry soil in kg.

The soil texture test was done by keeping the soil sample in to flask with water stirring the mixture well and waiting for some time. Once the larger particles of soil settled and formed layers based on size and density, the soil texture class was identified using a soil texture triangle.

To measure soil pH level a Tabletop pH meter (HI 2210) was used which has a scale of 0–14. Most plants thrive in less acidic to neutral (pH 6.0 to 7.0). The SEC was measured in a laboratory using a JENWAY 4310 conductivity meter. The CEC is the property of the soil's capacity to draw in, hold onto, and exchange cation elements in meq/100g of soil which is tested according to the NCR-13 standard. Based on Walkley and Black's standard protocol, the proportion of SOC and SOM was measured using the chromic acid method and gravimetric weight change method respectively. The alkaline permanganate method was used to assess the amount of available STN.

## 3 Results and discussion

### 3.1 Soil composition

**Fig. 4:**
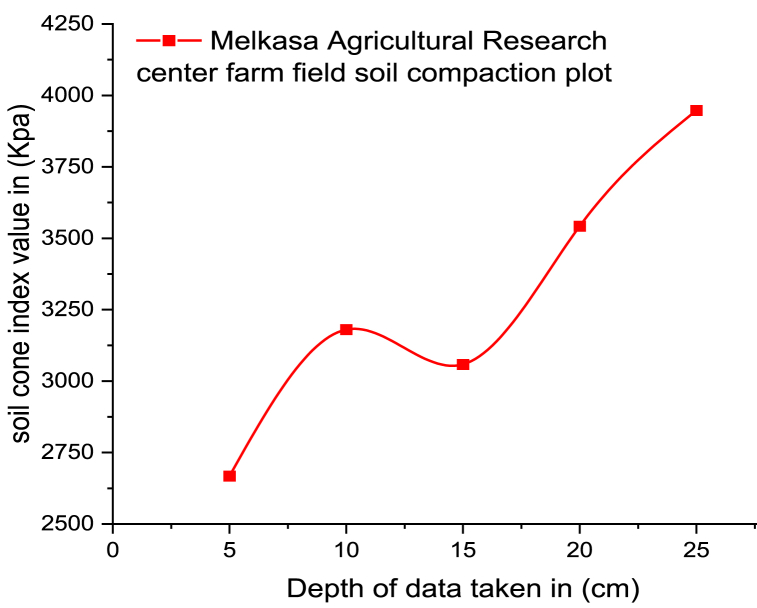
All sample points average soil cone index vs. depth.

The soil compaction test results for the experimental farm field are indicated in Fig. 4 taking average value for all sample points in the field from A to O. Soil compaction was increased as the test depth increased except for 10 cm–15 cm depth. The relation between soil compaction and depth were studied and the result of those researchers support this paper's result [[25], [26], [27]].

The correlation of sample points A to O soil compaction in the experimental farm field shows both positive and negative relations as indicated in Table 1.

**Table 1:** Sample points soil compaction correlation.

	A	B	C	D	E	F	G	H	I	J	K	L	M	N	O
A	1														
B	.444	1													
C	.523	.912	1												
D	−.568	−.003	−.36	1											
E	.915	.725	.775	−.52	1										
F	−.672	−.764	−.68	.282	−.879	1									
G	.452	.679	.530	.293	.466	−.292	1								
H	.827	.672	.853	−.72	.934	−.747	.334	1							
I	.752	.203	.080	−.21	.666	−.697	.165	.410	1						
J	.599	.647	.381	.298	.622	−.635	.798	.337	.666	1					
K	.773	.890	.842	−.17	.891	−.765	.798	.795	.466	.793	1				
L	.930	.558	.707	−.73	.964**	−.780	.294	.963^b^	.625	.424	.767	1			
M	.176	.706	.872	−.28	.408	−.253	.444	.612	−.402	.030	.565	.381	1		
N	.447	.709	.685	.077	.491	−.240	.943^a^	.478	−.031	.596	.800	.372	.692	1	
O	.468	.903^a^	.950	−.17	.672	−.522	.735	.731	−.029	.465	.864	.573	.897^a^	.871	1

a At the 0.05 level of significance.

b At the 0.01 level of significance, the letter ‘A to O indicates the sampling point in fields.

The field test sample points L and E, L, and H exhibit strong positive correlation at a 0.01 significant level. Sample points O and B, N, as well as G, O, and M, show a strong positive correlation at the 0.05 significant level. Generally, within this study field, there is a significant difference in soil compaction.

### 3.2 Physiochemical properties of soil

The laboratory test results of soil physical and chemical characteristics are summarized in Table 2.

**Table 2:** The laboratory result of soil physical and chemical properties for the farm field.

Depth	pH	SEC (μS/cm)	Textural			Textural class	SCEC (meq/100 g soil)	% STOC	% SOM	% STN	% Moisture
			% Sand	% Clay	% Silt						
0–10	7.28	113.3	37	29	34	CL	18.78	1.21	2.09	0.13	13.97
10–20	7.42	118.25	37	31	32		22.1	1.43	2.15	0.19	14.35
20–30	7.12	126.5	36	31	33		28.6	1.18	2.12	0.17	16.04
**Average**	**7.27**	**119.35**	**36.0**	**30.3**	**33**		**23.16**	**1.27**	**2.12**	**0.16**	**14.79**

Abbreviations: SEC = Soil electrical conductivity, SCEC = Soil cation exchange capacity, STOC = Soil organic carbon, SOM = Soil organic matter, STN = Soil total nitrogen, CL = clay loam.

SCEC is the ability of soil to store nutrients such as NH₄⁺, H, Ca, Mg, K, and Na [28,29]. The SCEC test result indicates 23.16 meq/100 g soil from samples tested as an average. As per the agrotechnical requirement standard, the farm soils have SCEC of 10–40 meq/100 g soil. So, this study result was in the range of agrotechnical requirement standards. The laboratory test result of moisture content indicates a 14.79 % average. The class of soil texture was clay loam soil with individual values of 33 % silt soil, 30.3 % clay soil, and 36.7 % sand soil as per the soil texture triangle [18]. The maximum amount of SCEC indicates that a higher amount of clay percentage has greater water-holding capacity and maximum organic matter in the soil [30].

**Fig. 5:**
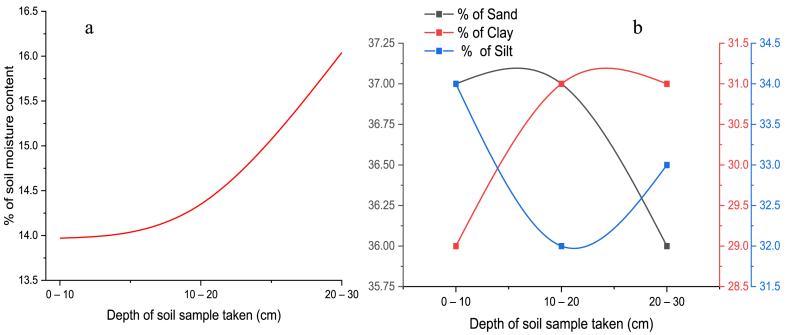
a) Moisture content of the soil, b) Sand, Clay, and Silt percent vs. depth.

The soil physiochemical properties that influence soil compaction are plotted in Fig. 5 to Fig. 7. As indicated in Fig. 5a the soil moisture content increases polynomial as the depth increases. As per the objective of this study, the impact of soil moisture content on the soil compaction is visible in Fig. 4, Fig. 5a. When the farm field soils have a high amount of water content, they become exposed to compacting easily. So, based on Figs. 4 and 5a both soil penetration resistance or soil compaction and soil moisture content are increased with the depth. This study produced comparable results to a previous study conducted by Refs. [31,32]. The soil compaction and moisture content result of this study is also supported by other researchers [33]. The highest and lowest value moisture were 13.97 % and 16.04 % respectively.

As Fig. 5b shows the silt and sand percentage was decreased with depth up to 20 cm and the clay percentage increased throughout the experimental test depth range. After 20 cm the sand and clay soil percentage start to decline but, the silt percentage starts to increase as indicated in Fig. 5b. In this research paper, the effect of soil texture on soil compaction was studied. As per the result of the experiment clay soil has more impact on soil compaction and also has a positively linear relation with that of soil compaction. The higher clay percentage of soil indicates more SCEC and organic matter in the soil which has a greater chance for soil compaction [30].

**Fig. 6:**
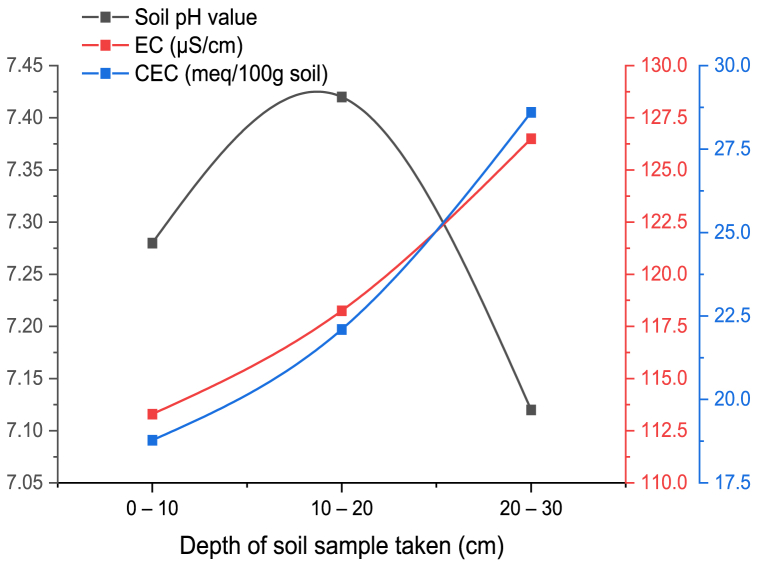
Soil PH, SEC, and SCEC vs. depth.

The fertility of soils decreases with decreasing pH by acidifying nitrogen amount in the soil, and leaching of the nitrate in the farming practices [34]. The minimum value of CEC of soil, the earlier the soil pH reduces with time. The soil pH value was increased with depth up to the depth of 20 cm. as indicated in Fig. 6. The SEC and SCEC have a positive relationship throughout the experiment test with soil compaction and depth as shown in Fig. 4, Fig. 6. But after 20 cm depth soil pH decreased and this conclusion was validated by Refs. [31,35].

**Fig. 7:**
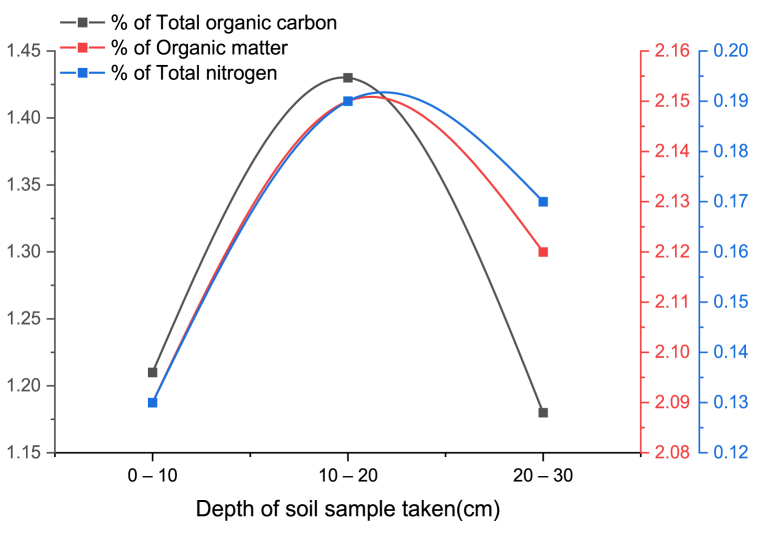
STOC, SOM, and STN percentage vs. depth.

Agricultural soils depend heavily on soil organic matter, which accounts for only 2–10 % of the mass of the majority of soils. Changes in soil organic carbon concentration affect the physical properties of the soil. Macro aggregation is encouraged by an increase in soil organic carbon, which also lowers the likelihood of soil compaction and improves water retention. Aggregates are formed by the combination of soil particles with binding agents produced by organic components [36]. As part of organic residues, elements like nitrogen, phosphorus, sulfur, potassium, calcium, and magnesium are also present in trace levels in soil organic matter [37].

In the experimental farmland, this study demonstrated a positive association between SOC, SOM, and STN with soil compaction and depth on the top part of the soil up to a depth of 20 cm as indicated on Figs. 4 and 7. After 20 cm depth, SOC, SOM, and STN start decreasing as shown in Fig. 7. Generally, the result of this research was supported by other related literature and they showed the SOC, SOM and STN increased with soil compaction and depth at the fertile depth of soil and decreased at the non-fertile depth [38].

## 4 Conclusions

Soil compaction test was taken at five different depths with the help hydraulic powered a hydraulic-powered Spot-on digital soil cone penetrometer. The soil texture and moisture content among soil physical properties and soil chemical characteristics like pH value, SEC, SCEC, SOM, STN, and SOC were determined in a laboratory. The highest and the minimum values of soil compaction were 6159 Kpa and 327 Kpa at sample points A and F, respectively. Taking average value for all sample points the highest and lowest soil compaction values were 3947.3 Kpa and 2667.7 Kpa at 25 cm and 5 cm depth, respectively. Soil compaction was increased as the test depth increased except for 10 cm–15 cm depth. The SCEC test result indicates 23.16 meq/100 g soil from samples tested as an average. The laboratory test result of moisture content indicates a 14.79 % average. The class of soil texture was clay loam soil with individual values of 33 % silt soil, 30.3 % clay soil, and 36.7 % sand soil as per the soil texture triangle. Both soil compaction and soil moisture content are increased with the depth. The highest and lowest value moisture were 13.97 % and 16.04 % respectively. The result of the experiment shows clay soil has more impact on soil compaction. The higher clay percentage of soil indicates more SCEC and organic matter in the soil which has a greater chance for soil compaction. The SEC and SCEC have a positive relationship throughout the experiment test with soil compaction and depth. In the experimental farmland, this study demonstrated a positive association between SOC, SOM, and STN with soil compaction and depth on the top part of the soil up to a depth of 20 cm. After 20 cm depth, SOC, SOM, and STN start decreasing.

## Funding statement

This research did not receive any specific grant from funding agencies in the public, commercial, or not-for-profit sectors.

## Data availability statement

Data will be made available on request through correspondent author.

## Ethics approval

Not applicable.

## Consent for publication

Not applicable.

## CRediT authorship contribution statement

**Yared Seifu Woldeyohannis:** Conceptualization, Data curation, Formal analysis, Investigation, Methodology, Software, Validation, Writing – original draft, Writing – review & editing. **Someshakher S Hiremath:** Supervision, Writing – review & editing. **Simie Tola:** Supervision, Writing – review & editing. **Amana Wako:** Supervision, Writing – review & editing.

## Declaration of competing interest

The authors declare that they have no known competing financial interests or personal relationships that could have appeared to influence the work reported in this paper.
